# Advances in the Degradation of Polycyclic Aromatic Hydrocarbons by Yeasts: A Review

**DOI:** 10.3390/microorganisms12122484

**Published:** 2024-12-02

**Authors:** Francisco Padilla-Garfias, Minerva Araiza-Villanueva, Martha Calahorra, Norma Silvia Sánchez, Antonio Peña

**Affiliations:** Departamento de Genética Molecular, Instituto de Fisiología Celular, Universidad Nacional Autónoma de México, Circuito Exterior s/n, Ciudad Universitaria, Mexico City 04510, Mexico; maraiza@ifc.unam.mx (M.A.-V.); mcalahor@ifc.unam.mx (M.C.); nsanchez@ifc.unam.mx (N.S.S.)

**Keywords:** polycyclic aromatic hydrocarbons, biodegradation, yeasts, bioremediation, xenome

## Abstract

Polycyclic aromatic hydrocarbons (PAHs) are toxic organic compounds produced during the incomplete combustion of organic materials and are commonly found in the environment due to anthropogenic activities such as industrial and vehicular emissions as well as natural sources, mainly volcanic eruptions and forest fires. PAHs are well known for their bioaccumulative capacity and environmental persistence, raising concerns due to their adverse effects on human health, including their carcinogenic potential. In recent years, bioremediation has emerged as a promising, effective, and sustainable solution for the degradation of PAHs in contaminated environments. In this context, yeasts have proven to be key microorganisms in the degradation of these compounds, owing to their ability to metabolize them through a series of enzymatic pathways. This review explores the advancements in yeast-mediated degradation of PAHs, with a particular focus on the role of enzymes such as cytochrome P450 (CYPs), epoxide hydrolases (EHs), and glutathione S-transferases (GSTs), which facilitate the breakdown of these compounds. The review also discusses the applications of genetic engineering to enhance the efficiency of yeasts in PAH degradation and the use of omics technologies to predict the catabolic potential of these organisms. Additionally, it examines studies addressing the degradation of benzo[a]pyrene (BaP) by yeasts such as *Debaryomyces hansenii*, and the potential future implications of omics sciences for developing new bioremediation.

## 1. Introduction

**Figure 1:**
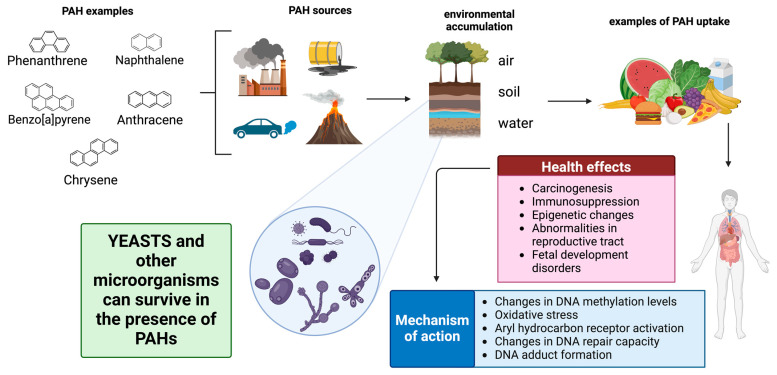
Schematic representation of the general characteristics of polycyclic aromatic hydrocarbons (PAHs) and their interaction with humans and microorganisms. Examples of PAHs are shown, which are generated by anthropogenic activities or natural phenomena, and, due to their physicochemical properties, tend to accumulate in the environment. When humans contact these compounds, for instance, through food, and metabolize them, they can pose health risks. Yeasts isolated from PAH-contaminated sites are proposed as a bioremediation strategy. Created with BioRender.com/p83a541 (accessed on 15 October 2024).

In recent decades, environmental contamination by hazardous substances has become a critical global concern, largely due to the anthropogenic input of polycyclic aromatic hydrocarbons (PAHs) into ecosystems, which far exceeds natural sources. PAHs, produced primarily by the incomplete combustion of organic materials, are highly persistent and toxic pollutants [1]. These environmental threats have driven the search for novel bioremediation methods. While microorganisms, such as bacteria, filamentous fungi, and microalgae, have been extensively studied for their role in biodegradation, advances in yeast research have lagged behind those on other microorganisms, despite initial studies dating back to the 1960s [2,3]; however, they have recently attracted attention for their potential in this area [4,5,6] (Figure 1).

Several species of yeasts from the Ascomycota and Basidiomycota phyla have been isolated from hydrocarbon-contaminated environments [7]. Research has shown that these yeasts possess intracellular enzymatic systems capable of degrading a wide range of xenobiotics. Despite their promising potential, the specific role of yeasts in PAH biotransformation remains largely unexplored. Their capacity for degradation appears to depend on the ecological niche and nutritional conditions of the microorganisms [3]. As current physicochemical remediation methods, such as incineration, base-catalyzed dechlorination, UV oxidation, and solvent extraction, often exacerbate pollution, yeast-mediated biodegradation is an increasingly attractive alternative [8].

The application of advanced genetic, genomic, proteomic, and metabolomic techniques, along with genetic engineering, has significantly improved our understanding of the physiology, ecology, and biochemistry of PAH-degrading microorganisms. Even now, researchers are developing novel PAH metabolic pathways by recombining diverse catabolic genes from various organisms into a single host cell through genetic manipulation [3,9]. This is particularly important for PAHs trapped in coal tar or soot particles, which impede remediation due to reduced bioavailability. To address this challenge, microorganisms must excrete biosurfactants to enhance the bioavailability of organic pollutants and prevent the accumulation of toxic intermediates [10].

Despite significant advances, substantial progress is still needed to achieve effective PAH removal across different environmental contexts. The microbiome of contaminated ecosystems plays a pivotal role, and understanding its metabolic processes could provide key insights into their xenome (the members of the detoxification pathways in the cell [11]) [3]. This knowledge could guide the development of targeted bioremediation strategies.

PAH-degrading fungi are divided into two main groups: ligninolytic and non-ligninolytic fungi. Ligninolytic fungi are those capable of secreting enzymes that degrade lignin and other compounds related to cellulose. In this review, we will focus on yeasts, which are mostly considered to be non-ligninolytic fungi. However, recent studies have isolated and identified yeast strains capable of utilizing lignin that are resistant to phenolic compounds [3,12,13,14].

Yeasts can transform PAHs through poorly understood metabolic pathways (Figure 1). The ongoing research seeks to further elucidate these mechanisms to enhance bioremediation efforts [6,9]. The objective of this review is to present a synthesis of the most significant contemporary issues in the research on PAH degradation by yeasts, highlighting the current challenges and future research directions.

## 2. Polycyclic Aromatic Hydrocarbons (PAHs)

### 2.1. Definition and Physicochemical Properties

Polycyclic aromatic hydrocarbons (PAHs) are non-polar organic compounds composed of fused aromatic rings without heteroatoms or substituents. Their molecular structure grants them chemical stability and resistance to many chemical and biological agents, allowing them to persist in the environment for long periods [9,15]. This structural resilience, attributed to their resonance energy, is also responsible for their hydrophobic nature, which further contributes to their persistence (Figure 1). While this stability is advantageous in industrial applications requiring chemical and thermal stability, it represents substantial challenges in terms of environmental degradation [15].

PAHs exhibit unique spectroscopic properties, which are useful for their identification. These compounds possess characteristic ultraviolet–visible (UV-Vis) absorption spectra and emit fluorescence under UV radiation, due to their π-electron systems, facilitating their identification in environmental samples [15,16]. Additionally, some large PAHs demonstrate semiconducting behavior, which is linked to their extended electronic structures [16].

PAHs are solid, colorless to slightly yellowish compounds with a faint odor. They have low water solubility, high lipophilicity, and relatively high melting and boiling points, and low vapor pressure. Their physicochemical properties, including solubility, volatility, and persistence, depend on the number of fused aromatic rings [8]. Low-molecular-weight PAHs (two or three rings), such as naphthalene, are more volatile and soluble. In contrast, high-molecular-weight PAHs (four or more rings), such as benzo[a]pyrene (BaP), are less soluble, more persistent, and toxic, with significant carcinogenic potential [16,17].

PAHs are found in air, water, soil, and sediments (Figure 1), accumulating primarily in soil, where they can persist for decades. This persistence has led to the development of physicochemical remediation guidelines in several countries [8,9]. However, remediation often merely redistributes contaminants between environmental compartments, such as from soil to water [8].

The environmental persistence of PAHs is closely related to their molecular structure. Low-molecular-weight PAHs are more volatile and easier to detect [1]. Conversely, studies by Dell’Anno et al. reveal that the addition of *Aspergillus* sp. (filamentous fungi) to marine sediments accelerates the degradation of high-molecular-weight PAHs more than that of low-molecular-weight PAHs, indicating preferential biodegradation [18].

### 2.2. Sources of PAH Contamination

PAHs are introduced into the environment through both natural and anthropogenic sources. Natural sources include volcanic eruptions and forest fires. However, most PAH emissions arise from human activities, particularly the incomplete combustion of fossil fuels such as coal, oil, and gas, as well as industrial activities, transportation, and domestic heating. Additional sources include vehicle emissions, cigarette smoke, grilling of meat over charcoal, the production of chemicals such as tar, asphalt, and creosote, and industrial processes like petroleum refining and the manufacture of plastics, paints, and pesticides (Figure 1) [1,19].

### 2.3. Toxicity, Human Health Risks, and Environmental Persistence

Due to their lipophilicity, PAHs tend to accumulate in the fatty tissues of organisms, particularly in aquatic ecosystems, and may enter the food chain, increasing the risk of exposure to top predators, including humans. Bioaccumulation of PAHs in fish and shellfish is a significant concern in industrial and urban areas, and in some regions, their consumption is regulated [20]. PAHs are known carcinogens, mutagens, and teratogens. For instance, BaP is especially toxic and has been shown to be epigenotoxic, neurotoxic, teratogenic, and harmful to fertility [21]. Chronic exposure to PAHs can affect the hematological, immune, and reproductive systems, as well as fetal developmental (Figure 1) [17,21,22].

### 2.4. Regulation and Remediation

Once PAHs are introduced into food, no effective method exists for their elimination. Consequently, prevention strategies focus on limiting the release of PAHs into the environment, especially from industrial sources. In food safety, the European Food Safety Authority (EFSA) employs the Margin of Exposure (MOE) approach to assess PAH risks in food. An MOE above 10,000 is considered safe, while values equal to or below this threshold are deemed risky [23].

The degradation of PAHs in the environment highly depends on the presence of specific microorganisms, namely, bacteria, fungi (including yeasts), and microalgae, and environmental factors, such as pH, temperature, and oxygen levels, that influence the rate of degradation [9]. Bioremediation strategies have proven to be effective, but require careful management to avoid transfer of contaminants between environmental compartments. Integrated approaches combining physical separation and biological degradation are under investigation to address PAH contamination in soil, water, and air [1,15,20].

## 3. Yeast-Mediated PAH Degradation

Yeasts offer a promising approach for the remediation of environments contaminated by PAHs. Unlike physical and chemical methods, bioremediation utilizes the natural ability of microorganisms to transform pollutants into less toxic compounds [3,9,24,25,26].

Yeasts, as eukaryotic organisms widely distributed in nature, have key environmental applications, including the removal of heavy metals from wastewater and the degradation of high-molecular-weight PAHs, e.g., BaP (Figure 1) [5,6,27,28]. Mycoremediation, a subcategory of bioremediation focusing on fungi (including yeasts), has proven effective in degrading recalcitrant PAHs, particularly in extreme environments where other microorganisms, such as bacteria, might not survive [4,5,6,29,30]. This technique can be applied either in situ, directly at the contamination site, or ex situ, in controlled facilities that optimize microbial activity, although the latter is usually more expensive [31,32,33].

Several yeast species, such as *Candida guilliermondii*, *Candida lipolytica* (in older articles it is common to find *C. lipolytica* but it is important to note that the name was updated to *Yarrowia lipolytica* due to taxonomic revisions [34,35]), *Candida maltosa*, *Candida tropicalis*, *Candida viswanathii*, *Cryptococcus* spp., *Debaryomyces hansenii*, *Exophiala* spp., *Hanseniaspora opuntiae*, *Hanseniaspora valbyensis*, *Pichia anomala*, *Rhodotorula* spp., *Saccharomyces cerevisiae*, *Torulopsis* spp., *Trichosporon* spp., and *Wickerhamiella* spp. have shown high efficacy in degrading high-molecular-weight PAHs [4,5,6,25,26,36,37,38,39,40,41,42,43]. Table 1 provides an overview of studies conducted over the past 60 years using yeasts for PAH degradation (adapted and updated from Padilla-Garfias et al. [27]).

These yeasts not only survive in contaminated environments but also efficiently degrade compounds like BaP, reducing their toxicity and transforming them into less harmful products, such as 2-hydroxymuconic semialdehyde, 3-hydroxy-benzo[a]pyrene, 9-hydroxy-benzo[a]pyrene, hydroxy-naphthoic acid, phthalic acid, phthalic anhydride, and others [5,36]. The activity of key intracellular enzymes, such as cytochrome P450 (CYPs), epoxide hydrolases (EHs), and glutathione S-transferases (GSTs), are directly linked to their ability to degrade PAHs into these less toxic metabolites [3,9].

It is important to mention that in bacteria, ligninolytic fungi, and microalgae there are other multi-enzyme systems, such as laccase or dioxygenases, which have rarely been described in yeast [3,9,25,26]. These multi-enzymatic systems involved in the degradation of PAHs and other xenobiotics have been referred to by experts like Edwards et al. [56] as the “xenome”, a term that has since been adopted by other authors [3,6,13,27].

## 4. Mechanisms of PAH Degradation by Yeasts

It has been reported that yeasts degrade PAHs through metabolic processes that involve a series of intracellular enzymatic reactions. However, prior to intracellular degradation, some yeasts, such as *Y. lipolytica*, have the ability to produce biosurfactants that enhance the solubilization and bioavailability of PAHs [51,55].

**Figure 2:**
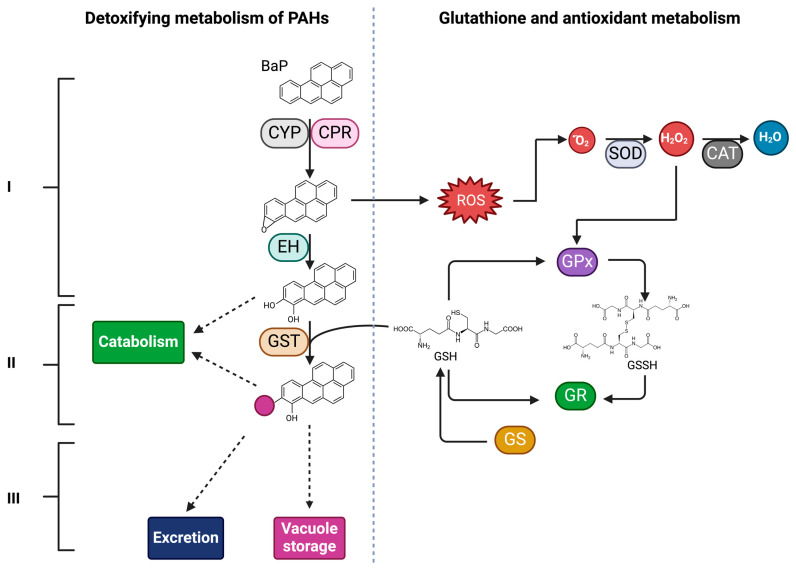
Detoxification and degradation system of benzo(a)pyrene (BaP) in yeasts. Phase I: BaP is transported into the cell, where it is transformed by enzymes such as cytochrome P450 monooxygenase (CYP), accompanied by cytochrome P450 reductase (CPR), which donates the necessary electrons to CYP, and epoxide hydrolase (EH). Phase II: the compound is hydroxylated by glutathione-S-transferase (GST), forming conjugated compounds (the pink circle represents glutathione (GSH)), which are less toxic and more soluble. Phase III: excretion of the conjugated compound, which is less toxic than unmetabolized BaP or internalization into the vacuole, and assimilation by other organisms. During BaP degradation, there are changes in GSH homeostasis due to GST activity, leading to the activation of antioxidant enzymes. This system ensures effective degradation and detoxification of BaP, as well as neutralization of reactive oxygen species (ROS), thereby protecting the cell from oxidative damage. Created with BioRender.com/u85w889 (accessed on 15 October 2024).

In this review, we will focus on the intracellular enzymatic mechanisms described in yeast, mainly on metabolism, which is traditionally categorized into three phases. These metabolic pathways are typically divided into three main stages: the initial oxidation of the compound (phase I), its subsequent degradation into less toxic products (as already discussed) or conjugation via transferase enzymes (phase II), and the excretion for utilization by other organisms, or storage of the conjugated metabolites into the vacuole (phase III) [3,13,57] (Figure 2). Phase I is crucial, as PAHs, due to their stable aromatic structure, cannot be directly broken down by yeasts without prior activation [3,13]. This activation process is catalyzed by enzymes such as CYPs [6,24,58], which oxidize the aromatic rings of PAHs, converting them into epoxides. Epoxides are reactive intermediates more easily metabolized in the subsequent phases of the degradation process [59].

### 4.1. Biosurfactant Production

Biosurfactants are surface-active compounds synthesized by yeasts and bacteria that are used to emulsify hydrophobic hydrocarbons such as PAHs, thereby reducing interfacial energy. They are usually classified into glycolipids, lipopeptides, and fatty acids, and are biodegradable, non-toxic, and effective under extreme conditions [60]. Common types of biosurfactants include non-ionic biosurfactants, such as ethoxylates, and ionic biosurfactants, such as fatty acids and quaternary ammonium salts [61].

Some microorganisms enhance hydrocarbon biodegradation by using biosurfactants and extracellular enzymes that increase the solubility of hydrocarbons and facilitate their adhesion to cells [62,63]. Active enzymes, such as alkane hydroxylase and oxygenases, work synergistically to degrade contaminants in complex environments [61].

Studies have demonstrated the potential of *Candida* species, such as *C. tropicalis*, *C. glabrata*, and *Y. lipolytica*, to remove hydrophobic compounds in oil bioremediation treatments, highlighting the simultaneous effect of cell adhesion and the production of biosurfactants and emulsifiers [51,55,64]. Optimizing this application requires a better understanding of adsorption and solubilization mechanisms in soil for future bioremediation applications [65].

### 4.2. Enzymes Involved in Degradation

The efficiency of yeast in degrading PAHs largely depends on the activity of certain intracellular enzymes which catalyze the initial stages of breakdown by facilitating the opening of aromatic rings and the subsequent conversion of intermediates into less hazardous compounds. Some of the most studied enzymes in yeast are outlined below.

#### 4.2.1. Cytochrome P450 (CYP) and Its Role in Epoxidation

CYPs are hemoproteins that catalyze hydroxylation, epoxidation, and monooxygenation reactions. They play crucial roles in the biosynthesis of secondary metabolites, ergosterol, sporogenesis, and the degradation of xenobiotics such as PAHs. It is important to mention that CYP proteins require a reductase to donate electrons in order to carry out oxidation. These reductases are known as cytochrome P450 reductases (CPRs) [58].

CYPs are one of the primary enzymes responsible for the activation of PAHs in yeast cells (Figure 2), catalyzing the epoxidation of aromatic rings, which generates epoxides [66]. The epoxidation process is essential because PAHs, in their non-reactive form, are highly stable and resistant to degradation. Introducing oxygen into the PAH structure, CYPs create reactive sites that can be targeted by other enzymes, facilitating their breakdown [58].

The ability of yeasts to express CYPs in response to the presence of PAHs is a key factor in determining their effectiveness in bioremediation. Without this enzyme, PAHs would remain stable and persist in the environment, limiting the yeast’s capacity to degrade them [9]. Studies have shown that genetically modified yeasts overexpressing CYPs can degrade PAHs far more efficiently than unmodified strains, suggesting that genetic engineering is a valuable tool for enhancing bioremediation [6,67].

The action of CYPs is considered one of the most important reactions in PAH degradation, as demonstrated by Padilla-Garfias et al. (2022) in *D. hansenii* [6], which, like that of *Aspergillus* spp. [68], facilitates the opening of PAH aromatic rings, resulting in the corresponding epoxide.

#### 4.2.2. Epoxide Hydrolases (EHs) and Their Role in Hydrolysis

EHs are enzymes that catalyze the hydrolysis of electrophilic epoxides (Figure 2), which are typically genotoxic, transforming them into the corresponding diols that are less reactive, more soluble, and less toxic than the original epoxides [69]. This process is essential for reducing the toxicity of these highly reactive and mutagenic compounds [7]. EHs are ubiquitous enzymes, found in the cytosol or microsomes, depending on the species, and participate in the detoxification of xenobiotics, as well as in the synthesis of chemical messengers and secondary metabolites [70].

Their role in the degradation of PAHs is particularly significant, as EHs not only reduce the toxicity of the compounds, but also facilitate their further degradation by other enzymes or their removal from the yeast cells. The hydrolysis of epoxides is, therefore, a critical step in the bioremediation of PAHs [3,9].

#### 4.2.3. Glutathione S-Transferases (GSTs)

GSTs are broadly distributed in the fungal kingdom; these enzymes are cytosolic, mitochondrial, and microsomal proteins that increase the solubility of compounds by forming various conjugates. They are also involved in defense mechanisms against reactive oxygen species through their thioltransferase or peroxidase activities [3]. Their main function is the conjugation of reactive compounds with glutathione (GSH), a tripeptide that functions as a detoxifying agent [13,57,71]. By conjugating epoxides and diols with GSH, GSTs facilitate their excretion from the organism and prevent the accumulation of toxic compounds within cells [3] (Figure 2).

GSTs play a key role in preventing the formation of reactive intermediates that could cause damage to DNA and other biomolecules [72]. Through the conjugation of these compounds with GSH, GSTs protect cells from the toxic effects of PAHs and their intermediates, which is essential for ensuring the safety of the bioremediation process [73].

#### 4.2.4. Other Relevant Enzyme Systems

In addition to the principal phase I and II enzymes, other enzyme systems play a substantial role in PAH degradation by yeasts, either by directly contributing to PAH degradation or by supporting the cellular processes necessary for efficient degradation. Among these, lignin peroxidases (LiPs) and manganese peroxidases (MnPs), traditionally associated with ligninolytic fungi, have demonstrated their potential in yeast species such as *R. mucilaginosa* and *Y. lipolytica* [2,5,20,74,75]. These enzymes oxidize aromatic compounds and destabilize their structures, although their activity in yeast appears to be species-specific and dependent on environmental context [20,76].

In a transcriptomic study conducted on the basidiomycetous yeast *R. mucilaginosa*, open reading frames (ORFs) encoding potential extracellular enzymes such as laccases and peroxidases were identified, similar to those of ligninolytic fungi. These enzymes can also oxidize PAHs prior to CYP activity. However, when enzymatic activity assays were performed, no activity was detected [5].

Alcohol dehydrogenase and alcohol oxidase are another important group of enzymes that catalyze the oxidation of the alcohol groups of PAH metabolites to aldehydes. This step is essential for subsequent degradation by aldehyde dehydrogenases (ADHs), which convert aldehydes to carboxylic acids [2,5,15,20,77]. These products are less toxic and can be integrated into cellular metabolism [15,77]. In *Rhodosporidium kratochvilovaeya* (syn, *Rhodotorula kratochvilovae*), these pathways channel metabolic intermediates into the tricarboxylic acid (TCA) cycle or lipid synthesis, highlighting their role in contaminant degradation and energy metabolism [26,78,79].

Esterases, although underexplored in yeasts, hydrolyze complex PAH derivatives, especially ester-bound contaminants in environmental matrices, thereby increasing their bioavailability for further enzymatic processing [75]. In addition, dioxygenases, such as catechol 1,2-dioxygenase and catechol 2,3-dioxygenase, catalyze the cleavage of aromatic rings after initial oxidation, producing intermediates such as cis-dihydrodiols and catechols that are essential for subsequent degradation [5,15,75,80].

Some authors suggest that phase I enzymes generate reactive oxygen species (ROS), leading to oxidative stress that activates the antioxidant defense system, which includes enzymes such as superoxide dismutase (SOD), catalase (CAT), and glutathione peroxidase (GPx), along with non-enzymatic compounds like GSH. Since GSH is also the substrate for GSTs, high GST activity may alter GSH metabolism, prompting the involvement of other enzymes like glutathione reductase (GR) or glutathione synthetase (GS) [5,81] (Figure 2). This has been observed in *Ulva lactuca* (Chlorophyta) when exposed to BaP and in *Aspergillus sydowii* (a filamentous fungus) after exposure to BaP and phenanthrene [81,82].

Finally, carbohydrate transferases (glycosyltransferases) and sulphate transferases (sulphotransferases), like GST, are key players in the conjugation and detoxification of PAH metabolites [3,5,27]. By adding sugar or sulphate groups to hydroxylated intermediates, these enzymes increase solubility, facilitating excretion or sequestration in the vacuole [3,75,83]. In *Y. lipolytica*, glycosylation has been suggested to play a dual role in detoxification and cellular signaling, highlighting its importance in yeast physiology and bioremediation [84,85]. In a transcriptomic study carried out in *R. mucilaginosa* in the presence of phenanthrene and BaP, upregulation of various transferases such as glutathione, sulphate, and carbohydrate transferases were detected [5].

The diversity of enzymatic systems employed by yeasts highlights their metabolic adaptability in degrading PAHs. However, the limited activity of some enzymes under laboratory conditions highlights the critical role of environmental factors, cofactors, and regulatory mechanisms in modulating their expression and activity. Future research should focus on elucidating these regulatory networks and using genetic engineering approaches to enhance enzymatic activity, paving the way for more efficient bioremediation strategies [86].

The study of mechanisms related to the emulsification and metabolism of hydrocarbons has been crucial in understanding how certain yeast strains can survive, resist, and thrive in environments contaminated with hydrocarbons. This knowledge has been used to improve the ability of certain strains to withstand exposure to hydrocarbons, as we will discuss below.

## 5. Metabolites Formed During Degradation

During the degradation of PAHs by yeasts, various intermediate and final metabolites are produced, some of which may exhibit varying degrees of toxicity [5,7,24,81]. The identification and characterization of these metabolites are crucial for assessing the effectiveness and safety of bioremediation processes [3,5,9,81].

As previously mentioned, epoxides and diols can be more toxic than the original PAHs, making the subsequent degradation steps essential for reducing harmfulness of the generated metabolites [3,9,25]. As stated above, in *R. mucilaginosa* it has been reported and determined the formation of products such as 2-hydroxymuconate semialdehyde, 3-hydroxy-benzo[a]pyrene, 9-hydroxy-benzo[a]pyrene, hydroxy-naphthoic acid, phthalic acid, phthalic anhydride, among others, resulting from the degradation of BaP [5].

Patel et al., (2017) described a detailed metabolic pathway of how the oleaginous yeast *Rhodosporidium kratochvilovaeya* degrades phenol, a non-polycyclic aromatic hydrocarbon, finding that its metabolites could be incorporated into Krebs cycle and lipid metabolism, as they identified metabolites such as succinate, acetyl CoA, formate, acetaldehyde, and pyruvate [78].

One of the challenges in mycoremediation is ensuring that the products formed during the degradation process are not more toxic than the original compounds [5,81]. Consequently, research in mycoremediation has focused not only on enhancing the efficiency of PAH degradation but also on ensuring that intermediate products are rapidly converted into less harmful compounds through the coordinated action of different enzymes [10].

Several authors have investigated the toxicity of metabolites formed after the degradation of PAHs in culture media supernatants. The results indicate that these supernatants are not toxic to the moss *Physcomitrium patens* (formerly *Physcomitrella patens*), neither do they affect the bioluminescence of the bacterium *Aliivibrio fischeri*. In addition, no damage was observed in mammalian cells such as erythrocytes and the lung epithelial cell line A549 [5]. Similar tests with *A. sydowii* showed consistent results [81].

Monitoring these metabolites is essential to evaluate the safety and effectiveness of the mycoremediation process, as some intermediate metabolites may possess mutagenic or toxic properties if not adequately managed [5].

## 6. Genetic Engineering and Biotechnology

The application of genetic engineering has revolutionized the field of bioremediation by enhancing the degradative capacities of yeasts [87]. Commonly used tools such as cloning, along with cutting-edge technologies like CRISPR-Cas9 (already utilized in white-rot fungi, filamentous fungi, yeasts, and oomycetes) [88,89,90] have enabled precise and efficient modification of genes involved in the expression of essential enzymes for PAH degradation, such as CYPs [58,87,91].

One of the most promising approaches has been the genetic engineering of yeast strains to overexpress CYPs, enabling higher rates of PAH and xenobiotic degradation [6,49,92,93]. This results in the accelerated activation of recalcitrant compounds, speeding up their degradation [49,92]. Furthermore, genetic modification has facilitated the development of yeasts capable of tolerating higher concentrations of PAHs [6] and other contaminants, making them ideal for use in highly polluted environments. In areas where natural conditions limit microbial activity, these modified strains present an effective solution for remediating contaminated sites [87].

Genetic engineering has also facilitated the incorporation of genes from other microbial species into yeasts, broadening their capacity to degrade a wider range of toxic compounds [6,49,92,93]. For instance, genes encoding monooxygenases of aromatic compounds, commonly found in fungi that thrive in the presence of lignin, have been introduced into yeasts to enhance their ability to degrade high-molecular-weight PAHs, which are particularly difficult to break down [49]. This approach has enabled yeasts to degrade not only BaP but also more complex PAHs.

In the future, CRISPR-Cas9, a tool that allows precise genetic manipulation, may be employed to further optimize yeast strains for bioremediation, maximizing their efficiency in degrading environmental pollutants [88,89,90]. Additionally, modifying regulatory genes could optimize the conditions under which degradative enzymes are expressed, further enhancing the bioremediation process. This underscores the importance of ongoing scientific research in this field [94].

Notably, the heterologous expression of a novel MnP encoded by the *pimp1* gene, was successfully achieved in *S. cerevisiae*. This gene, derived from the white rot fungus *Peniophora incarnata* KUC8836, demonstrates the capability to degrade anthracene. The enzyme encoded by the *pimp1* gene was secreted by the yeast into the culture medium, exhibiting a maximum activity of 3.58 U/mL [95].

Recently, Padilla-Garfias et al. (2022) [6] identified *D. hansenii* as one of the most promising yeast species for BaP degradation, comparing its degradation rates with those of *C. albicans*, *R. mucilaginosa*, and *S. cerevisiae*. In this study, *D. hansenii* was shown to degrade BaP through a CYP enzyme encoded by the *DhDIT2* gene. When a specific inhibitor of this enzyme was added, BaP degradation did not occur. Results demonstrated that *D. hansenii* degraded over 80% of the BaP in the experiment (100 ppm) in the absence of the inhibitor, without compromising cell viability, even under different temperature conditions. The study also utilized genetic engineering, heterologously expressing the *DhDIT2* gene in two *S. cerevisiae* strains, one lacking the *ScDIT2* gene and another possessing its own *ScDIT2*. The strain lacking *ScDIT2* could neither tolerate nor degrade BaP, but when complemented with the *DhDIT2* gene, it regained its ability to grow in the presence of BaP and to degrade it. The strain with both *ScDIT2* and *DhDIT2* showed an increased degradation capacity and was able to tolerate BaP at lower temperatures. This research demonstrated that the efficiency of *S. cerevisiae* in degrading BaP could be enhanced through genetic engineering by expressing, alongside its own, the *D. hansenii*’s CYP [6].

These advancements suggest that genetically modified strains of *S. cerevisiae*, *D. hansenii*, and other organisms could be used effectively in industrial processes that emit PAHs or other xenobiotics, improving efficiency and reducing the time needed to clean up contaminated environments. In this context, it is proposed that these industries include in their processes a preliminary bioremediation step using genetically engineered strains for rapid biodegradation of pollutants. Subsequently, the controlled inactivation of the organisms used is suggested, and finally the release of the waste, which will no longer contain toxic compounds. This approach would allow safer and more sustainable management of industrial waste [96,97,98].

In practical terms, the ability of *D. hansenii* to degrade BaP holds significant implications for bioremediation. Field studies have shown that this yeast can be used in situ to treat PAH-contaminated soils and waters, a particularly relevant feature due to its marine origin [6]. Furthermore, *D. hansenii* also has the advantage of being more resistant to extreme conditions such as low temperature or high salinity [99,100,101].

As genetic engineering technology continues to advance, the prospects for improving the degradative capacities of yeasts become increasingly promising. Genetically modified yeasts (GMOs) could play a key role in implementing sustainable and efficient solutions for cleaning up PAH-contaminated sites, particularly in industrial and urban areas where these compounds are more prevalent [96].

While GMOs have greatly improved the degradative capabilities of organisms such as yeasts, it is essential to implement appropriate regulatory measures to govern their use. We propose the strategic utilization of GMOs as tools for the heterologous expression of genes linked to the degradation of hazardous aromatic pollutants (HAPs). This method allows researchers to explore the functions of enzymes encoded by these genes and to characterize them in greater detail [97,98].

## 7. Omics Tools and PAH Degradation

**Figure 3:**
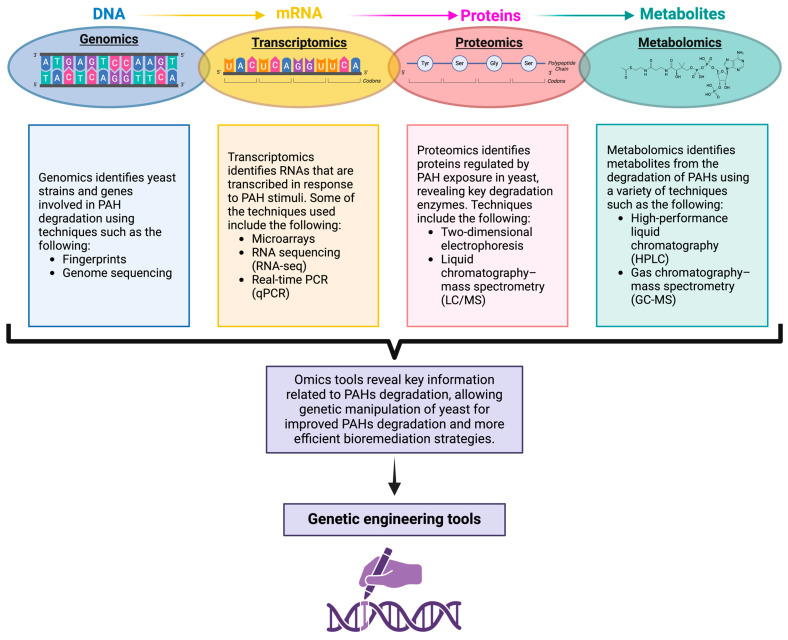
Omics tools and their relationship with genetic engineering to enhance yeast for the degradation of polycyclic aromatic hydrocarbons (PAHs). Omics tools, such as genomics, transcriptomics, proteomics, and metabolomics, allow the identification of key genes and metabolic pathways in yeasts, facilitating their manipulation through genetic engineering to design more efficient routes for mycoremediation. Created with BioRender.com/q09y374 (accessed on 15 October 2024).

Omics tools, including genomics, transcriptomics, proteomics, and metabolomics, provide valuable insights into the metabolic pathways involved in the degradation of pollutants such as BaP. These techniques are interdependent and complementary, offering a comprehensive means to explore degradation pathways (Figure 3).

Genomics has provided critical information into the molecular mechanisms underlying PAH biodegradation by yeasts [5]. Advances in genome sequencing and omics techniques have enabled the identification of genes responsible for PAH degradation, facilitating the development of optimized strains for bioremediation [3,9] (Figure 3). Recent studies utilizing fingerprinting and pyrosequencing technologies have consistently shown that fungi thrive in a variety of PAH-contaminated environments, including soil, rhizospheric seagrass sediment, water bodies, streams, and even extreme habitats such as polluted soils in Antarctica and burned landscapes, among others [102,103,104,105,106,107].

Molecular technologies have revealed that the primary contributors to polluted environments are predominantly from the phylum Ascomycota and the subphylum Mucoromycotina, with a lesser representation from the phylum Basidiomycota [3]. The predominant fungal species in biostimulated aged creosote soil belong to the genera *Alternaria* sp., *Chaetomium* sp., *Neurospora* sp., and *Fusarium* sp. [102].

At present, functional genomic approaches are being utilized to investigate the connections between genotype and phenotype, as well as the metabolic processes that facilitate PAH transformation, although the latter is less commonly examined. The enzymes responsible for intracellular detoxification pathways are encoded by multigene families (as has been reported for the genes coding for CYP and GST [57,58,71]) that constitute the xenome [13]. The xenome, then, is the biosystem responsible for detecting, transporting, and metabolizing xenobiotics [56].

Metagenomics, a branch of genomics, allows the study of the genetic material of microorganisms in their natural environment, using sequencing techniques to analyze the genomes of all the organisms in a sample without isolating individual species [108], and enables the study of microbial genomes in specific environments, such as soils or waters contaminated with PAHs, without the need to culture individual microorganisms [109]. This method has revealed entire microbial communities contributing to PAH degradation, including previously unrecognized species, such as *Rhodotorula* sp. and *Exophiala* sp., identified in petroleum-contaminated sites in Mexico [4]. Studies have shown that specific genes correlate with the ability of microbes to thrive in PAH-polluted environments, offering valuable ideas for engineering more effective microbial consortia for bioremediation in situ [110].

Through metagenomic analysis, co-metabolic pathways of pyrene degradation have been proposed, shedding light on the microbial degradation. The proposed pathways involve oxidation, dioxygenation, and ring cleavage, with specific enzymes such as PAH dioxygenases playing a crucial role in the initial oxidation step. Additionally, metagenomics has highlighted symbiotic interactions between microbial species, leading to more integrated bioremediation strategies [111].

For instance, bacteria unable to degrade high-molecular-weight PAHs can support yeasts by providing essential metabolites or altering the chemical environment to enhance yeast enzymatic activity. These microbial interactions improve overall bioremediation efficiency, potentially accelerating the degradation of contaminants [109]. As an example, metagenomic and genomic analyses have significantly contributed to the understanding of the degradation pathways, the microbial communities involved, and candidate genes participating in the degradation of BaP, providing valuable information for the management of pyrene contamination [109].

Transcriptomics examines all RNA molecules transcribed at a given time, revealing gene activity and responses to environmental stimuli. To achieve this, transcriptomics employs various technologies, including DNA microarrays, reverse transcription quantitative polymerase chain reaction (RT-qPCR), and RNA sequencing (RNA-seq), to investigate gene expression and the regulation of transcription [112] (Figure 3).

In PAH bioremediation, transcriptomic analyses have shown how yeasts react to pollutants, identifying genes that are induced for PAH degradation [5]. However, there have been few transcriptomic studies on fungi involved in PAH conversion, and these investigations are often tied to the availability of annotated genomes. For instance, microarrays developed from the model basidiomycete *P. chrysosporium*, a ligninolytic fungus, have demonstrated that CYPs are differentially regulated in response to anthracene, exhibiting distinct catalytic properties against both anthracene and anthrone [113]. In this species, the GSTs Ure2p4 and Ure2p6 show specific expression following PAH treatment [114]. Additionally, experiments using RT-PCR under non-ligninolytic conditions revealed that two key PAH-oxidizing P450 monooxygenases, CYP63A2 and CYP5136A3, were upregulated in the presence of BaP, resulting in the production of P450-hydroxylated metabolites [115].

Other studies highlight increased expression of genes encoding enzymes such as CYPs, EHs, and GSTs when yeasts are exposed to PAHs. Additionally, genes related to heat shock, oxidative stress, DNA protection, and detoxification are regulated according to the contaminant concentration, suggesting these enzymes as crucial for yeast survival in polluted environments [5], as found in some species of the filamentous fungus *Aspergillus* sp. [68,81]. This knowledge could aid in the optimization of yeast strains for enhanced PAH degradation, ensuring efficient enzyme expression in contaminated settings [87].

In contrast, studies on native Ascomycota have predominantly focused on pathogenesis and industrially relevant enzymes, while transcriptional research related to PAH conversion remains limited. This highlights the need for further investigations using transcriptional methods; nonetheless, there have been reports of upregulation of xenome genes in pollution-adapted fungal species [3,9,12].

Metabolomic studies have been particularly effective in identifying intermediate metabolites formed during PAH degradation by yeasts, such as diols and carboxylic acids, using techniques like high-performance liquid chromatography (HPLC) [5,36] and gas chromatography–mass spectrometry (GC-MS) [6] (Figure 3). These methods have mapped the complete metabolic pathways of PAH degradation. For instance, in *R. mucilaginosa*, metabolites such as phthalic acid derivatives and dihydroxybenzene, which integrate into benzoate degradation pathways, were identified during BaP oxidation, along with GSH and sulphate conjugates [5]. Metabolomics also tracks toxic intermediates, allowing researchers to optimize experimental conditions or genetically modify strains to improve the efficiency and safety of bioremediation [111].

Furthermore, proteomics has revealed key enzymes involved in PAH degradation and detoxification by identifying those that increase in response to exposure and prioritizing key enzymes (Figure 3). Traditionally, the exploration of enzymatic systems has relied on metabolite detection and protein purification techniques. However, advancements in proteomic technologies are transforming this landscape, particularly in the context of fungal responses to environmental pollutants like PAHs [9].

The field of proteomics, which initially utilized two-dimensional (2D) electrophoresis, is now significantly enhanced by the integration of liquid chromatography coupled to mass spectrometry (LC/MS). This advancement provides a powerful suite of tools for assessing the metabolic adaptations of fungi that enable them to flourish in PAH-contaminated environments. Next-generation proteomics employs sophisticated mass spectrometric separation techniques, including quadrupole, time-of-flight (TOF), and linear ion trap (Orbitrap™) mass analyzers. These technologies facilitate the accurate quantification of protein expression through label-free methods and stable isotope labelling, such as isobaric tags for relative and absolute quantitation (iTRAQ) [116].

Current research has predominantly focused on the extracellular proteome, particularly the secretome of fungi known for their cellulolytic and ligninolytic capabilities, due to their significant biotechnological applications [117]. In contrast, proteomic studies investigating PAH transformation and catabolic pathways in Ascomycota are relatively scarce. This scarcity is largely attributable to the fact that key transformation processes occur within the mycelium and specific organelles. The niche area of ‘subproteomics’ [118] is emerging as a vital field, yet research on the regulation of microsomal protein expression remains nascent [117].

Notable studies, such as those conducted by Verdin et al., [119], utilized 2D gel electrophoresis to demonstrate the overexpression of CYPs in *Fusarium solani*, a filamentous fungus, in the presence of BaP, highlighting the ecological implications of this response. Additional investigations into the biodegradation of alachlor (a recalcitrant aromatic herbicide) by *Paecilomyces marquandii* (a filamentous fungus) and the degradation of 4-nonylphenol (a surfactant and pesticide, that disrupts the endocrine system) by *Metarhizium robertsii* (a mitosporic fungus) have identified various proteins associated with oxidative stress responses. These studies suggest that there may be unidentified proteins involved in the transformation of xenobiotics [120,121].

In *S. cerevisiae*, proteomic analysis showed that BaP exposure induces the production of heat shock proteins and other stress-related proteins, indicating that yeast survival in contaminated environments depends not only on enzymatic degradation but also on its ability to manage cellular stress [40].

As the field of fungal proteomics continues to expand, it holds the potential to elucidate the pathways involved in the conversion of harmful compounds, thereby informing the development of more efficient bioremediation strategies. Nevertheless, further research using shotgun proteomics is essential to advance our understanding in this area.

## 8. Conclusions

In recent years, research into the degradation of PAHs by yeasts has made significant strides, offering profound insights into the molecular mechanisms underlying this process and the practical applications of these microorganisms in bioremediation. Key enzymes, like CYPs, EHs, and GSTs, that comprise the xenome, play a central role in the breakdown of PAHs, transforming recalcitrant and hazardous compounds into less toxic and more manageable products, such as phthalic acid and its derivatives.

Advances in genetic engineering and omics techniques have markedly enhanced the capabilities of yeasts to degrade PAHs, resulting in the development of more efficient and resilient strains that can thrive in a variety of contaminated environments. Techniques such as metagenomics, transcriptomics, proteomics, and metabolomics have yielded a comprehensive understanding of the molecular and metabolic interactions that empower yeasts to break down PAHs, paving the way for innovative and more effective bioremediation strategies.

Looking forward, research is anticipated to focus on the identification of novel yeast strains with enhanced PAH degradation abilities, as well as the development of microbial consortia capable of synergistically degrading a broader range of organic pollutants in industrial and urban areas. Additionally, the application of genetically modified strains engineered to withstand extreme environmental conditions holds promise for expanding bioremediation applications, particularly in industrial waste management prior to environmental release. The integration of yeasts into bioremediation strategies represents a critical tool for addressing the environmental challenges of the 21st century, contributing significantly to sustainability and public health.

Understanding the role of yeasts in PAH degradation, alongside the future applications of omics science, not only addresses pressing concerns about the impacts of these compounds on health and the environment but also facilitates the development of sustainable and scalable remediation solutions. These advances underscore the indispensable role of microorganisms, such as yeasts, in promoting environmental sustainability, aligning with the United Nations (UN) Sustainable Development Goals (SDGs). Consequently, biotechnology-driven bioremediation is poised to emerge as a cornerstone technology for building a cleaner and safer future [122].

## Figures and Tables

**Table 1:** Main studies on yeast related to PAH degradation.

Year	Study
1965	The ability of *S. cerevisiae* to absorb BaP through passive diffusion was investigated [44].
1970s	The activity of BaP hydroxylase, later identified as CYP, in *S. cerevisiae* was examined [45]. A fluorometric assay was developed to measure BaP metabolites, revealing that CYP synthesis is regulated by cyclic AMP and influenced by glucose in the medium. It was further reported that BaP induces genetic activation of CYPs [46].
1981	The ability of *C. lipolytica* (later identified and characterized as *Y. lipolytica* [35]), *C. tropicalis*, *C. maltosa*, *C. guilliermondii*, and *D. hansenii* to oxidize naphthalene, biphenyl, and BaP was investigated, along with an early metabolomic analysis proposing the structures of some metabolites [36].
1982	The purification of CYP (also known as BaP hydroxylase) was performed to study its regulation and the metabolites produced when BaP was exposed to the enzyme [47,48].
1993	Yeast abundance was quantified in sediments from 13 coastal sites in Massachusetts, identifying genera such as *Candida* spp., *Cryptococcus* spp., *Rhodotorula* spp., *Torulopsis* spp., and *Trichosporon* spp. Over 50% of the isolates from contaminated areas transformed phenanthrene, with *Trichosporon penicillatum* exhibiting the greatest efficiency in transforming PAHs [37]. This work is considered the earliest metagenomic study.
2006	A strain of *P. anomala*, isolated from petroleum- and oil-contaminated soil, was characterized. This strain demonstrated the ability to degrade naphthalene, dibenzothiophene, phenanthrene, and chrysene [38].
2009	A strain of *C. viswanathii* capable of degrading a mixture of low- and high-molecular-weight PAHs, including naphthalene, phenanthrene, pyrene, and BaP, was isolated and characterized [39].
2010	Six CYP genes were identified in *Phanerochaete chrysosporium* and found to be induced by PAHs. These genes were cloned and expressed in *Pichia pastoris* alongside a reductase from *P. chrysosporium*, demonstrating oxidizing activity towards three-to-five-ring PAHs. The recombinant enzymes oxidized pyrene and BaP, enhancing *P. pastoris’* capacity to degrade PAHs [49].
2013	A functional toxicology proteomic analysis with *S. cerevisiae* identified the proteins required for cellular resistance to BaP by examining the activity of key genes involved in various stress response pathways, DNA repair, redox homeostasis, and oxidative stress [40].
2016	Four yeast strains, *D. hansenii*, *H. opuntiae*, *H. valbyensis*, and *Rhodotorula* sp., isolated from BaP-contaminated soils, were evaluated. In consortium studies, they achieved 76% degradation within 6 days under optimized conditions. Degradation products were identified, and a metabolic pathway involving several key enzymes was proposed [41].
2018	A yeast consortium composed of *D. hansenii*, *H. opuntiae*, *H. valbyensis*, and *Rhodotorula* sp., enriched with zinc oxide nanoparticles for the degradation of BaP in contaminated soils, was found to enhance degradation efficiency in the presence of the nanoparticles [42,43].
2020	Yeast strains of a novel anamorphic species were isolated from hydrocarbon-contaminated groundwater in Spain and a human infection in the USA. Phylogenetic analysis placed them in the *Wickerhamiella* clade, with *W. sorbophila* and *W. infanticola* as their closest relatives. The species *W. verensis* was proposed as new, with CECT 12028T as the holotype [50].
	Two fungal isolates from an oil-polluted site in Mexico were identified as a novel *Rhodotorula* sp. and *Exophiala* sp. Both strains showed pH and salinity tolerance, with *Exophiala* switching from hyphae to yeast at high salinity. *Rhodotorula* degraded single-ring aromatic hydrocarbons, while *Exophiala* removed polyaromatic hydrocarbons. Both strains grew well in the presence of aromatic compounds [4].
2021	*R. mucilaginosa* EXF-1630, isolated from Arctic Sea ice, was grown on phenanthrene and BaP under hypersaline conditions, achieving 80% removal in 10 days. Extracellular enzymes were undetected, but NADPH-cytochrome *c* reductase activity peaked at day 4. Non-toxic metabolites were confirmed, and transcriptomic analysis revealed extensive gene regulation in response to PAHs. This study is considered the first to describe a yeast’s metabolic profile and transcriptomic response to PAH degradation [5].
2022	*Cryptococcus albidus*, *C. guilliermondii*, and *C. tropicalis*, isolated from sugarcane, showed the ability to use lignin as the sole carbon source and to grow in the presence of phenol and its derivatives (pentachlorophenol and p-nitrophenol), with all strains exhibiting ligninolytic activity [14].
	The effect of BaP on the growth and metabolism of *C. albicans*, *D. hansenii*, *R. mucilaginosa*, and *S. cerevisiae* was evaluated. All species metabolized over 70% of BaP without affecting their viability, with *D. hansenii* showing the highest efficiency. The initial degradation step was found to be mediated by a CYP enzyme, and the *DhDIT2* gene in *D. hansenii* was identified as essential for this process. *D. hansenii* and *S. cerevisiae* expressing the *DhDIT2* gene are proposed as optimal candidates for BaP bioremediation in contaminated environments [6].
	*Y. lipolytica* IMUFRJ 50682, isolated in Brazil, efficiently degraded complex petroleum hydrocarbons, including n-alkanes, isoprenoids, and PAHs. In the process, it was found to produce biosurfactants [51].
	*D. hansenii* completely removed n-dodecane, a linear alkane, in saline effluent from desalination plants at 20 °C and 1–5 g/L salt, and demonstrated effectiveness in wastewater treatment in refineries [52].
2023	*S. cerevisiae*, *C. utilis*, and *Rhodotorula benthica* were used as exogenous organisms to treat soils contaminated with total petroleum hydrocarbons, improving degradation compared to that achieved by native microorganisms [53].
2024	*C. tropicalis* strain B isolated from hydrocarbon-contaminated seawater demonstrated a high capacity to degrade crude oil, with a wide tolerance of pH (4–11) and salinity (1–12%). With glucose and yeast extract, it enhanced its biodegradation capacity, reaching up to 98.6% removal of naphthalene and 79.48% of phenol [54].
	*Y. lipolytica* LMS 24B demonstrated high potential to produce biosurfactants capable of emulsifying hydrocarbons and metabolizing paraffin [55].
